# IL-1 Receptor Antagonist Protects the Osteogenesis Capability of Gingival-Derived Stem/Progenitor Cells under Inflammatory Microenvironment Induced by *Porphyromonas gingivalis* Lipopolysaccharides

**DOI:** 10.1155/2021/6638575

**Published:** 2021-01-16

**Authors:** Yuxin Zhao, Bobo Cai, Weijun Zhu, Jue Shi, Yu Wang, Misi Si

**Affiliations:** The Affiliated Hospital of Stomatology, School of Stomatology, Zhejiang University School of Medicine, and Key Laboratory of Oral Biomedical Research of Zhejiang Province, Hangzhou, Zhejiang 310006, China

## Abstract

Mesenchymal stem cells (MSCs) have been considered to be a future treatment option for periodontitis due to their excellent regenerative capability. However, it is still a challenge to protect MSCs' biological properties from multiple bacterial toxins in local inflammatory environment. The present study is aimed at investigating the treatment effect of interleukin-1 receptor antagonist (IL-1ra) on cell proliferation, migration, and osteogenic differentiation of gingival-derived mesenchymal stem cells (GMSCs) under an inflammatory microenvironment induced by *Porphyromonas gingivalis* lipopolysaccharides (*P. gingivalis-*LPS). GMSCs derived from Sprague-Dawley (SD) rats' free gingival tissues were treated with *P. gingivalis-*LPS (10 *μ*g/mL) to create *in vitro* inflammatory environment. Different concentrations of IL-1ra (0.01-1 *μ*g/mL) were used to antagonize the negative effect of LPS. Cell behaviors including proliferation, cloning formation unit (CFU), cell migration, osteogenic differentiation, mineral deposition, and cytokine production were assessed to investigate the protection effect of IL-1ra on GMSCs under inflammation. The toll-like receptor 4 (TLR4)/nuclear factor kappa B (NF-*κ*B) pathway activated by LPS was evaluated by real-time quantitative polymerase chain reaction (RT-PCR) and western blot. In response to *P. gingivalis-*LPS treatment, cell numbers, cloning formation rate, cell migration rate, proinflammatory cytokine production, and osteogenic differentiation-associated protein/mRNA expressions as well as mineralized nodules were suppressed in a time-dependent manner. These negative effects were effectively attenuated by IL-1ra administration in a time- and dose-dependent manner. In addition, mRNA expressions of TLR4 and IkB*α* decreased dramatically when IL-1ra was added into LPS-induced medium. IL-1ra also reversed the LPS-induced TLR4/NF-*κ*B activation as indicated by western blot. The present study revealed that IL-1ra decreased inflammatory cytokine production in a supernatant, so as to protect GMSCs' osteogenesis capacity and other biological properties under *P. gingivalis-*LPS-induced inflammatory environment. This might be explained by IL-1ra downregulating TLR4-mediated NF-*κ*B signaling pathway activation.

## 1. Introduction

Periodontitis, a highly prevalent inflammatory disease, is initiated by bacteria and their products and labelled by the progressive disintegration of alveolar bone. Previous studies have demonstrated that the onset and progression of periodontitis are correlated with *Porphyromonas gingivalis* (*P. gingivalis*), one of the keystone oral Gram-negative pathogens that can locally invade periodontal tissues [1, 2]. Lipopolysaccharide (LPS) is considered the major virulence factor of *P. gingivalis* in inflammatory response and periodontal tissue destruction [3]. Various periodontal therapies are performed; unfortunately, there still exist many obstructions in reversing bone resorption and regenerating lost periodontal tissues [4].

Mesenchymal stem cells (MSCs) play a critical role in bone regeneration by differentiation into osteogenic progenitor cells; therefore, they could be used as promising stem cell-based therapies in bone defects of periodontitis. Bone marrow stem cells (BMSCs) proved to own osteogenic effect in mandible critical-size defects when seeded on a bone porcine block in minipigs [4]. With appropriate scaffolds, human dental pulp stem cells (hDPSCs) could enhance in vivo bone formation [5, 6]. Gingival stem/progenitor cells (GMSCs), similar to BMSCs and hDPSCs, process self-renewal and multipotent differentiation potential. Zhang et al. [7] isolated progenitor/stromal cells from human gingival tissue for the first time and demonstrated their surface marker expression profile and multiple differentiation capacities. GMSCs are characterized by easy and minimally invasive isolation [8]. An elegant study by Tian et al. [9] has demonstrated that LPS-induced inflammation interfered with osteogenic differentiation and proliferation of the GMSCs. In this scenario, it is necessary to investigate effective therapy to protect GMSCs' ability in inflammatory condition.

An anti-inflammatory agent from a natural source could be a promising tool to restore the GMSCs' functions. IL-1 receptor antagonist (IL-1ra), as a natural antagonist to IL-1, inhibits IL-1*β* from binding to the IL-1 receptor and regulates IL-1 activity [10]. Zhang et al. [11] addressed that anti-inflammatory factors IL-1ra and TNF-*α* inhibitor (TNF-ai) could boost osteogenic differentiation potential of GMSCs. The fact that IL-1*α* reduced connective tissue attachment loss in experimental periodontitis supported the view that IL-1ra could play a vital role in controlling periodontal inflammation [12]. A previous elegant study concluded that in conjunction with IL-1ra-loaded HA hydrogel synthetic extracellular matrix (HA-sECM), the regenerative capacity of GMSCs could be enhanced in a typical periodontitis model in miniature pigs [13]. IL-1ra/G-MSCs/HA-sECM and G-MSCs/HA-sECM groups showed better histological, clinical, and radiological outcomes in periodontal tissue regeneration when compared to scaling and root planing (SRP) alone [13]. Compared with GMSCs/HA-sECM, the IL-1ra/GMSCs/HA-sECM group proved to exert significant improvement on periodontal attachment level, length of the junctional epithelium, connective tissue adhesion, cementum regeneration, and bone regeneration [13]. However, the underlying mechanism of IL-1ra as an anti-inflammatory agent on GMSCs in vitro has not been investigated.

Present studies proved that overexpression of IL-1ra could block the induction of the nuclear factor-kappa B (NF-*κ*B) pathway in Caco-2 intestinal epithelial cells [14]. A recent pilot clinical trial also illustrated that IL-1ra may decrease NF-*κ*B signaling in blood leukocytes, associated with prognosis of breast cancer [15]. The NF-*κ*B pathway is defined as a key factor to activate innate immunity; at the same time, it initiates osteoclast differentiation and terminal bone resorption in periodontitis [16–18]. Nevertheless, whether IL-1ra inhibits activation of the TLR4-mediated NF-*κ*B pathway in GMSCs remains uncertain.

In our present study, we isolated GMSCs from Sprague-Dawley (SD) rat gingival tissues as the research object. LPS from *P.gingivalis* was used to mimic an in vitro inflammatory milieu. Multiple concentrations of IL-1ra were added to the coculture model as antagonism. The aim of the study was to elucidate the contribution of IL-1ra on GMSCs' colony formation, proliferation, migration, and osteogenesis differentiation ability and to investigate proinflammatory cytokine secretion in inflammatory and anti-inflammatory conditions. Moreover, we detected the potential role of the distinctive TLR4-mediated NF-*κ*B signaling pathway in the processes.

## 2. Materials and Methods

### 2.1. Isolation and Culture of rGMSCs

This study was approved by the Animal Care and Use Committee of Zhejiang University with number ZJU20200085. The rGMSCs were isolated from healthy free gingival tissues of 6-week-old male SD rats. Briefly, rats were anesthetized and euthanized for the collection of gingival tissue around the molars. Gingival tissue was flushed with phosphate-buffered saline (PBS; HyClone, USA) and then detached, deepithelized, and minced into fragments. The tissue fragments were digested with dispase I (neutral protease; 4 mg/mL; Roche, USA) and collagenase I (3 mg/mL; Sigma-Aldrich, USA) for 2 hours and then maintained in *α*-minimal essential medium (*α*-MEM; HyClone) with 10% fetal bovine serum (FBS; Gibco (16000-044), USA) and 1% penicillin-streptomycin (ScienCell, USA) at 37°C in an incubator in 5% CO_2_. The medium was changed every 2-3 days. A limited dilution technique was used to obtain single-cell colony (passage 0). The cells from passage 2 to passage 5 were used for the subsequent experiments.

### 2.2. Flow Cytometric Analysis of the rGMSC Phenotype

Expressions of specific antigen on the rMSC surface were identified by flow cytometry analysis (FACS). Passage 3 rGMSCs were incubated at 4°C in the dark room for 30 min with FITC-conjugated monoclonal antibodies against rat STRO-1 (eBioscience, USA), CD34 (Abcam, UK), CD105 (Abcam), CD146 (Abcam), CD73 (Becton-Dickinson, USA), and CD90 (BioLegend, USA), with FITC-conjugated mouse anti-rat CD45 (Becton-Dickinson) and with FITC-conjugated rabbit CD14 (Bioss, USA). After thorough washing with PBS, cells were subjected to flow cytometry analysis to evaluate the proportion of expressions with the use of a FACScan flow cytometer (Becton-Dickinson).

### 2.3. Assessment of rGMSC Multilineage Differentiation Potential

- Osteogenic induction: rGMSCs from the second passages were seeded in six-well plates at 2 × 10^4^ cells/well until subconfluence for about twenty-four hours. For osteoblast differentiation, cells were incubated in osteogenic inductive medium containing 10 mmol/L *β*-glycerophosphate, 50 *μ*g/mL vitamin C, and 1 × 10^−7^ mol/L dexamethasone (all from Sigma) and then refreshed every three days. rGMSCs were also cultured at the same concentration in basal medium as a control. After 21 days for culture growth, Alizarin Red (Leagene, China) staining was performed to assess calcified deposition in both two groups. Calcium nodules of different wells were recorded with photos
- Adipogenic induction: rGMSCs from the second passages were seeded in six-well plates at the density of 2 × 10^4^ cells/well. When reaching confluence, adipogenic inductive medium supplemented with 10 mg/L insulin, 0.25 *μ*mol/L dexamethasone sodium phosphate, 0.5 mmol/L isobutyl methylxanthine, and 100 mmol/L indomethacin (all from Sigma) was added and then renewed every 3 days. For control, rGMSCs were cultivated at the same concentration in basic medium. Oil droplets in cells were stained with Oil Red O (Leagene) solution after 21 days. Formation of lipid-laden fat cells was observed by using an inverted microscope, and images were taken
- Chondrogenic induction: rGMSCs from the second passages were placed in six-well plates with the concentration of 2 × 10^4^ cells/well. After cell adhesion for 24 hours, chondrogenic inductive medium containing 10 *μ*g/L transforming growth factor- (TGF-) *β*1, 50 *μ*g/mL vitamin C, 1 × 10^−8^ mol/L dexamethasone, and 0.22 g/L sodium pyruvate (all from sigma) was added and then renewed every 3 days. After 21 days of cultivation, cartilage-specific proteoglycan was evaluated by Alcian blue (Solarbio) staining and recorded with photos

### 2.4. Experimental Groups

The rGMSCs from the second passages were trypsinized with 0.25% trypsin (Gibco), resuspended, and cultured under five different media. All of the media were replaced every 3 days. The experiment was divided into 5 groups:

- Negative control group (group 1): basic medium
- Inflammatory group (group 2): inflammatory medium consisting of *P. gingivalis*-LPS (10 *μ*g/mL) (InvivoGen, USA) in basic medium
- Anti-inflammatory group (group 3): supplemented with the anti-inflammatory cytokine IL-1ra (10 ng/mL) (PeproTech, USA) in inflammatory medium
- Anti-inflammatory group (group 4): supplemented with the anti-inflammatory cytokine IL-1ra (100 ng/mL) (PeproTech) in inflammatory medium
- Anti-inflammatory group (group 5): supplemented with the anti-inflammatory cytokine IL-1ra (1 *μ*g/mL) (PeproTech) in inflammatory medium

### 2.5. Colony-Forming Unit (CFU) Assay

To evaluate rGMSCs' colony-forming potential under inflammatory and anti-inflammatory conditions, the CFU assay was carried out. rGMSCs of different groups from the second passages were diluted and plated at 100 cells per well in a 6-well plate. After 14 days of incubation, cell cultures were washed with PBS, fixed with 4% paraformaldehyde (Solarbio), and then stained with GIEMSA (Solarbio) for 10 min. Aggregation of 50 or more cells was defined as a colony.

### 2.6. Cell Counting Kit-8 (CCK-8) Assay

The CCK-8 assay was conducted to measure cell viability and proliferation according to the manufacturer's instructions. rGMSCs from the second passages were inoculated on a 96-well plate at a density of 5000 cells/well. After cell adherence, the culture medium was replaced, respectively, according to group allocation outlined above. rGMSCs were incubated in normal, inflammatory, and anti-inflammatory medium for variable time intervals (1, 3, and 7 days). At the end of the culture period, CCK-8 (SAB, USA) solution was separately added and cells were incubated at 37°C for an additional 2 h. Finally, the optical density (OD) value at 450 nm was assessed by using a microplate reader (PerkinElmer/EnVision, USA).

### 2.7. Wound Healing Scratch Assay

rGMSCs from different groups were seeded in culture plates and were allowed to grow until 60%–80% confluence. After incubation, a 200 *μ*L pipette tip was used to generate a gap in the middle of the culture. Detached cells were gently removed with PBS. Fresh medium with mitomycin (Sigma) was added to each plate. After variable time intervals (24, 48, or 72 hours) of cell migration, the scratch wound closure was measured under an inverted microscope and analyzed with ImageJ software. The open area uncovered by rGMSCs in the culture expressed the results.

### 2.8. Osteogenic Differentiation Potential of Stimulated rGMSCs *In Vitro*

To evaluate the effect of inflammatory and anti-inflammatory cytokines on rGMSCs' osteogenic potential, osteogenesis-related gene, protein, and mineral deposition were separately assessed. After rGMSC adhesion, the medium was replaced with different osteogenic induction media according to group allocation for a total of 28 days of cultivation.

#### 2.8.1. Quantitative Real-Time Polymerase Chain Reaction (RT-PCR) Assay on Osteogenic Gene Expression

RT-PCR was used to qualify the mRNA expression levels of osteogenesis-related genes including alkaline phosphatase (ALP), osteopontin (OPN), osteocalcin (OCN), type I collagen (Col-I), and runt-related transcription factor 2 (Runx2). After variable time intervals (1, 7, and 14 days) of cultivation, total RNA was extracted from the cells by using the TRIzol reagent (Invitrogen, USA). Then, total RNA was subjected to reverse transcription to cDNA using the SuperScript First Strand Synthesis Kit (Fermentas, Japan) following the manufacturer's protocol. The real-time PCR analysis was conducted with SYBR Green PCR Kit (Thermo Fisher Scientific, USA) in a volume of 25 *μ*L reaction mixture, with 12.5 *μ*L of the SYBR Green Master Mix, 0.5 *μ*L of both forward and reverse primers, 2 *μ*L of specimen cDNA, and 9.5 *μ*L of ddH_2_O. All reactions were carried out for 10 minutes at 95°C, 40 cycles of 15 seconds at 95°C and 45 seconds at 60°C, 15 seconds at 95°C, 1 minute at 60°C, 15 seconds at 95°C, and 15 seconds at 60°C. *β*-Actin was selected as the internal control. Relative expressions of mRNA levels were calculated by the 2^−*ΔΔ*Ct^ method. The genes and primers used are listed in Table 1.

#### 2.8.2. Western Blot Analysis on Osteogenic Protein Expression

Western blot analysis was employed as a semiquantitative assay of the expression levels of ALP and OCN. The total proteins were extracted by using a radioactive immune precipitation assay (RIPA) (Solarbio) lysate from rGMSCs which had been incubated for 7 days and 14 days in different groups. To assure concentrations of protein from each sample, the bicinchoninic acid (BCA) method (Thermo) was used. Samples were then submerged in boiling water for 10 minutes. Proteins were separated by sodium dodecyl sulphate-polyacrylamide gel electrophoresis (SDS-PAGE) and transferred to polyvinylidene fluoride (PVDF) membranes (Millipore, USA). Subsequently, membranes were blocked with Tris Buffer Solution Tween (TBST) (5% nonfat milk, 0.1% Tween-20) and then incubated at 4°C overnight with the primary antibodies containing ALP (diluted to 1 : 1000; Abgent, AP13552a, USA), OCN (diluted to 1 : 5000; Abcam, ab133612, USA), and *β*-actin (diluted to 1 : 1000; Proteintech, 60008-1-Ig, USA). After full washing with TBST, samples were incubated with corresponding secondary antibodies (HRP-conjugated goat anti-rabbit IgG, A0208; diluted to 1 : 1000; Beyotime, China) at room temperature for 2 h. Immunoreaction was visualized by an enhanced chemiluminescence assay (ECL, PerkinElmer, USA), and the grey values of protein bands were quantified by using ImageJ.

#### 2.8.3. Extracellular Matrix Mineralization

Alizarin Red staining (ARS) was carried out to determine the effects of cytokines on mineral depositions. After 7, 14, and 28 days of induction, the cultures were fixed in 4% paraformaldehyde (Solarbio) for 30 minutes, stained with Alizarin Red (Leagene) for 30 minutes, and then desorbed with cetylpyridinium chloride (Aladdin, China) with continuous agitation. The absorbance at 560 nm of representative cultures was measured.

### 2.9. Detection of the Involvement of the NF-*κ*B Pathway in rGMSCs

#### 2.9.1. RT-PCR for NF-*κ*B Signaling mRNA Expression

RT-PCR was used to qualify the mRNA expression level of TLR4, an inhibitor of nuclear factor kappa-B alpha (I*κ*B*α*) at variable time intervals (1, 7, and 14 days). RT-PCR was performed in a similar way to the description in Section 2.8.1. Related genes and primers used are listed in Table 1.

#### 2.9.2. Western Blot Analysis for the NF-*κ*B Pathway

Western blot analysis was employed as a semiquantitative assay of the expression levels of NF-*κ*B p65, NF-*κ*B phosphorylated p65, I*κ*B*α*, and phosphorylated I*κ*B*α*. After incubation for 1 h, rGMSCs from different groups were washed with ice-cold PBS and then scraped off by using cell scrapers and sonicated for 10 seconds on ice. Western blot analysis was performed in a similar way to the description in Section 2.8.2. The primary antibodies used were as follows: NF-*κ*B p65 (diluted to 1 : 2000; Cell Signaling, 8242T, USA), NF-*κ*B phosphorylated p65 (diluted to 1 : 2000; Cell Signaling, 3033T), I*κ*B*α* (diluted to 1 : 2000, Affinity AF5002, USA), and phosphorylated I*κ*B*α* (diluted to 1 : 2000, Affinity AF2002).

### 2.10. Cytokine Release by Enzyme-Linked Immunosorbent Assay (ELISA)

rGMSCs were seeded at 7 × 10^4^ per well on six-well plates followed by variable time interval (12, 24, and 48 hours) stimulation according to the group allocation. The amounts of cytokines (IL-1*β*, TNF-*α*, and IL-1ra) presented in the supernatant were measured by using the ELISA Kit (Cusabio, China) following the manufacturer's recommendation. In brief, supernatants were collected and centrifuged (1000g at 4°C for 15 minutes). 20 *μ*L of each sample was pipetted into a 96-well plate and mixed with 20 *μ*L of the antibody cocktail (detector and capture antibodies) and then incubated for 2 hours. After washing and drying, 100 *μ*L substrate 3,3′,5,5′-tetramethylbenzidine substrate streptavidin alkaline phosphatase (Genzyme) was added to each well; then, the plates were incubated for 30 minutes in the dark on a plate shaker. Finally, stop solution was added and absorbance recorded at a wavelength of 450 nm by using an MCC 340 multiscan plate reader (Thermo Fisher Scientific Inc., Pittsburgh, PA).

### 2.11. Statistical Analysis

Summarized data are expressed as mean ± standard deviation (SD), and all measurements were conducted in triplicate. The Kolmogorov-Smirnov (K-S) test was performed to evaluate the normal distribution of variables. One-way analysis of variance (ANOVA) followed by the Fisher least significant difference (LSD) method was performed to test the significance of differences between the negative control group and all experimental groups. All analyses were performed by using SPSS software (SPSS version 19.0, Chicago, IL, USA), and photos were generated by using GraphPad Prism 7 software (GraphPad Software, Inc., La Jolla, CA, USA). *P* < 0.05 is considered statistically significant.

## 3. Results

### 3.1. Isolation and Identification of rGMSCs

**Figure 1:**
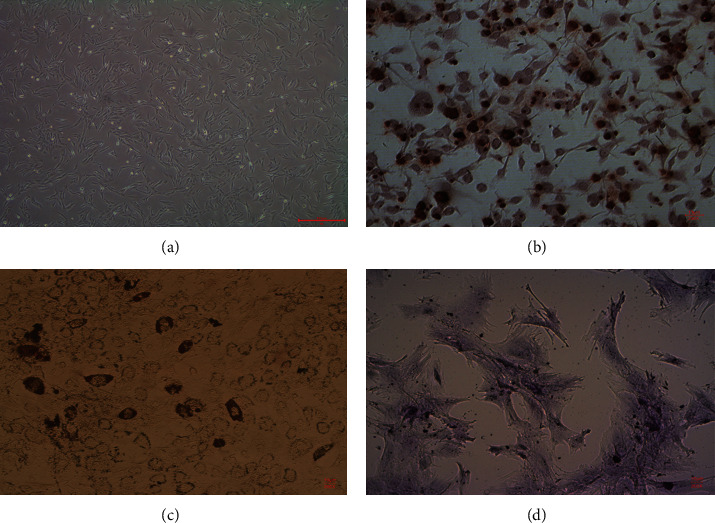
Characterization and multidirectional differentiation potential of stem/progenitor cells from rat gingival tissues. (a) Microscopic appearance of rGMSC morphology (20x). (b) Osteogenic differentiation of rGMSCs after 21 days of osteogenic induction. Positive staining of calcified deposits is observed (200x). (c) Adipogenic differentiation of rGMSCs after 21 days of adipogenic induction. Positive staining of lipid droplets is observed (200x). (d) Chondrogenic differentiation of rGMSCs after 21 days of chondrogenic induction. Positive staining of glycosaminoglycans is observed (200x).

**Figure 2:**
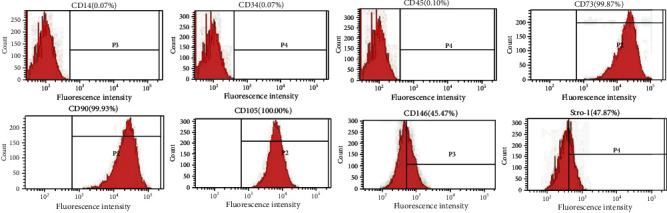
FACS identification of the surface marker expressions of rGMSCs. CD73, CD90, CD105, CD146, and Stro-1 were highly expressed, while rGMSCs were negative for CD14, CD34, and CD45.

After growing from rat gingival tissues, rGMSCs assumed fibroblast-like cluster morphology (Figure 1(a)). The Alizarin Red, Oil Red O, and Alcian blue staining demonstrated that rGMSCs owned the ability to differentiate into osteoblasts (Figure 1(b)), adipocytes (Figure 1(c)), and chondrocytes (Figure 1(d)). At the same time, calcified deposits formed in osteogenically induced rGMSCs, lipid droplets were detected in adipogenic differentiation of rGMSCs, and extracellular glycosaminoglycans deposited in chondrogenic-induced rGMSCs. Flow cytometry was used to analyze surface markers of rGMSCs. As shown in Figure 2, rGMSCs showed positive expressions for mesenchymal stem cell markers STRO-1, CD73, CD90, CD105, and CD146 and negative expressions for CD14, CD34, and CD45.

### 3.2. Colony Formation, Proliferation, and Migration of rGMSCs under Inflammatory/Anti-Inflammatory Environment

**Figure 3:**
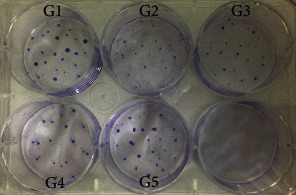
Colony formation of rGMSCs under inflammatory and anti-inflammatory environment. GIEMSA staining results for the colony-forming unit (CFU) assay (G1: GMSCs; G2: GMSCs+LPS; G3: GMSCs+LPS+10 ng/mL IL-1ra; G4: GMSCs+LPS+100 ng/mL IL-1ra; and G5: GMSCs+LPS+1 *μ*g/mL IL-1ra).

**Figure 4:**
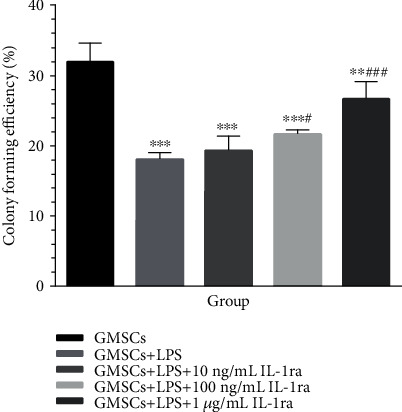
Effect of *P. gingivalis*-LPS and IL-1ra on rGMSC colony formation efficiency at 14 days. ^∗∗^*P* < 0.01, ^∗∗∗^*P* < 0.001 vs. GMSCs; ^#^*P* < 0.05, ^###^*P* < 0.001 vs. GMSCs+LPS.

After 14 days of incubation, colony formation efficiency in both the inflammatory group and anti-inflammatory groups was inhibited (Figures 3 and 4). *P. gingivalis*-LPS significantly suppressed rGMSCs' colony formation efficiency (*P* < 0.001). This inhibition could be partly reversed after coculturing with the anti-inflammatory cytokine IL-1ra. Anti-inflammatory groups displayed noticeable higher colony formation efficiency in a dose-dependent manner than the inflammatory group (*P* < 0.05). However, no statistically significant difference was found between the anti-inflammatory group with low IL-1ra (10 ng/mL) and the inflammatory group. All groups showed decreasing levels of colony formation compared to the control.

**Figure 5:**
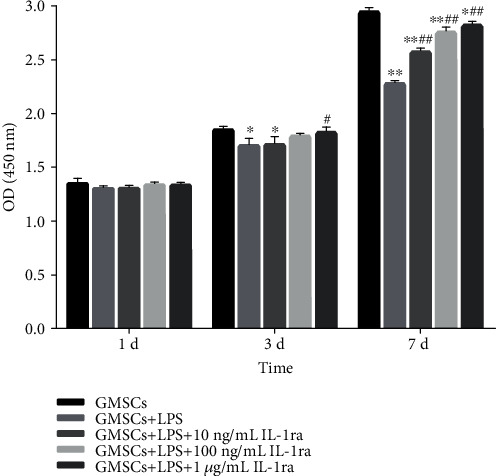
Effect of *P. gingivalis*-LPS and IL-1ra on rGMSC proliferation potential at 1, 3, and 7 days. ^∗^*P* < 0.05, ^∗∗^*P* < 0.01 vs. GMSCs; ^#^*P* < 0.05, ^##^*P* < 0.01 vs. GMSCs+LPS.

As shown in Figure 5, a similar trend was observed in cell viability by the CCK-8 assay. The OD values of rGMSCs decreased under *P. gingivalis*-LPS treatment, while they are gradually raised with increasing IL-1ra concentrations. After 24 hours of cultivation, a similar proliferation rate was detected in five groups. After 3 days of treatment, only the anti-inflammatory group with high IL-1ra (10 ng/mL) showed elevated level when compared to the inflammatory group (*P* < 0.05). After 7 days of cultivation, all IL-1ra-treated rGMSCs performed remarkably stronger proliferative ability in a dose-dependent manner than rGMSCs in the inflammatory group (*P* < 0.01). All groups showed decreasing levels of cell numbers compared to the control.

**Figure 6:**
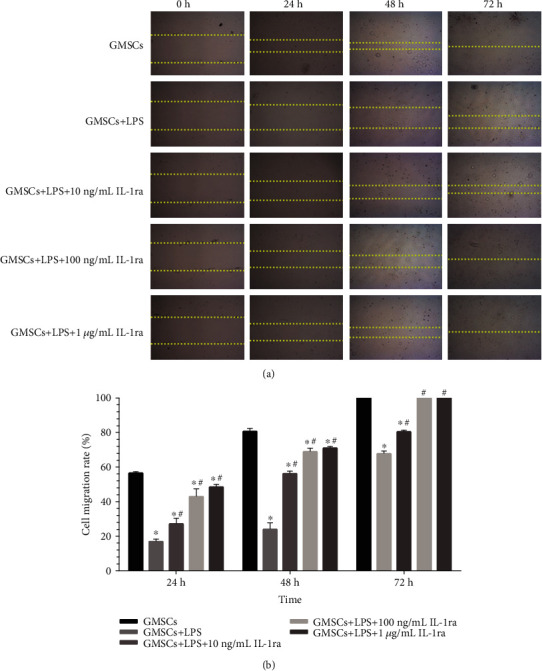
Effect of *P. gingivalis*-LPS and IL-1ra on rGMSC migration capacity at 24, 48, and 72 hours. (a) Migration of rGMSCs to cover the scratch area. (b) Cell migration rate in the control group, inflammatory group, and anti-inflammatory groups at different time points. ^∗^*P* < 0.05 vs. GMSCs; ^#^*P* < 0.05 vs. GMSCs+LPS.

The wound healing scratch assay was performed (Figure 6(a)), and the cell migration rate at different time points is shown in Figure 6(b). For all time intervals (24, 48, and 72 hours), the percentage of wound covered for rGMSCs with IL-1ra treatment was significantly higher than rGMSCs with *P. gingivalis*-LPS treated in a dose-dependent way (*P* < 0.05). At 72 hours after the scratch test, rGMSCs stimulated with 100 ng/mL or 1 *μ*g/mL IL-1ra fully filled the wound scratch area. All groups showed decreasing levels of cell migration rate at 24 and 48 hours compared to the control.

### 3.3. rGMSCs' Osteogenic Potential under Inflammatory Environment/Anti-Inflammatory Environment

**Figure 7:**
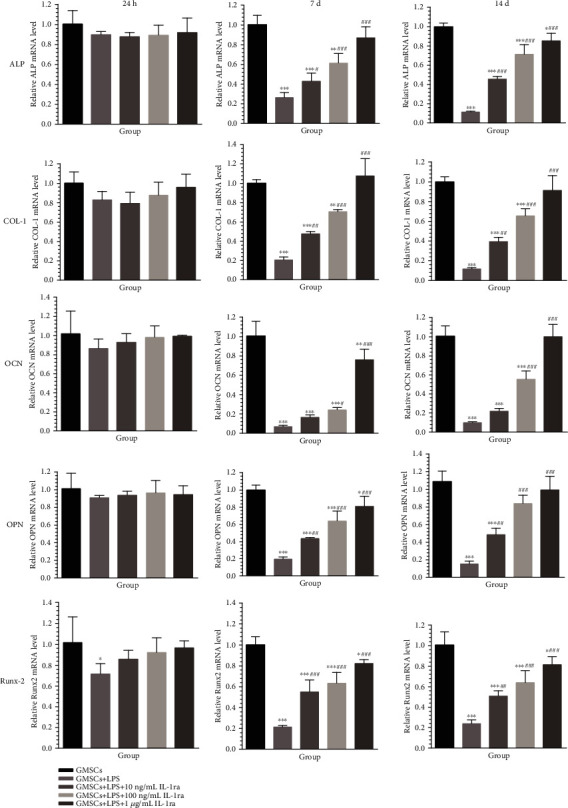
mRNA expression of osteogenic genes including ALP, COL-I, OPN, OCN, and RNX2 in different groups after 24 hours, 7 days, and 14 days of osteogenic induction, detected by RT-PCR. ^∗^*P* < 0.05, ^∗∗^*P* < 0.01, and ^∗∗∗^*P* < 0.001 vs. GMSCs; ^#^*P* < 0.05, ^##^*P* < 0.01, and ^###^*P* < 0.001 vs. GMSCs+LPS.

The mRNA expressions of osteogenic markers including ALP, COL-I, OPN, OCN, and RNX2 were measured by RT-PCR (Figure 7). There was no statistically difference between different groups after only 1 day of osteogenic induction. OCN expression in the anti-inflammatory group with 10 ng/mL IL-1ra did not show statistical increase in comparison with that in the inflammatory group. Other mRNA expressions of osteogenic-related genes clearly enhanced in a concentration-dependent manner in the presence of IL-1ra when compared with the inflammatory group (*P* < 0.01). Except COL-I (7 days) in the anti-inflammatory group with high IL-1ra (1 *μ*g/mL), other groups showed decreasing mRNA levels of osteogenic-related genes at 7 days and 14 days compared to the control.

**Figure 8:**
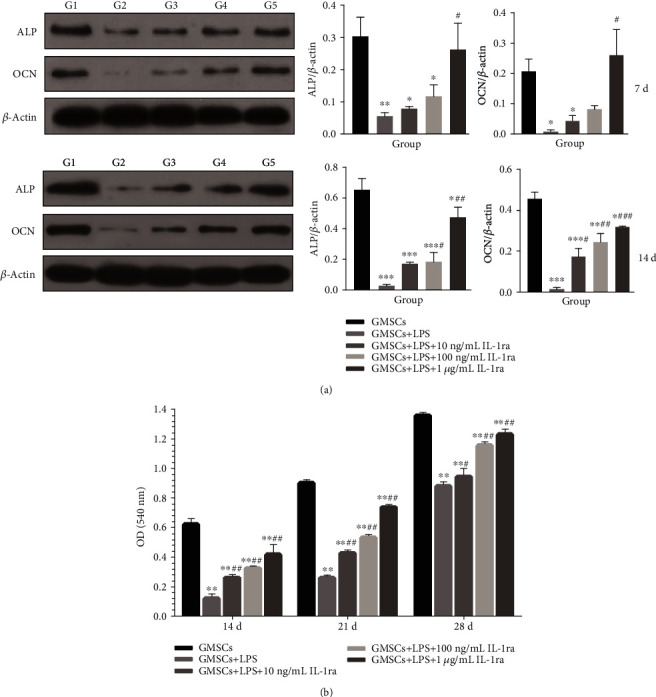
rGMSC osteogenic gene expression on protein level and calcified nodule deposition. (a) Expression of osteogenic-related proteins ALP and OCN in rGMSCs after 7 days and 14 days of osteogenic induction, detected by western blot (G1: GMSCs; G2: GMSCs+LPS; G3: GMSCs+LPS+10 ng/mL IL-1ra; G4: GMSCs+LPS+100 ng/mL IL-1ra; and G5: GMSCs+LPS+1 *μ*g/mL IL-1ra). (b) Mineralized nodule formation by rGMSCs after 14 days, 21 days, and 28 days of osteogenic induction, detected by Alizarin Red staining. ^∗^*P* < 0.05, ^∗∗^*P* < 0.01, and ^∗∗∗^*P* < 0.001 vs. GMSCs; ^#^*P* < 0.05, ^##^*P* < 0.01, and ^###^*P* < 0.001 vs. GMSCs+LPS.

These remarkable effects on osteogenic differentiation depending on *P. gingivalis*-LPS and IL-1ra dose were further confirmed by measurement of ALP and OCN proteins (Figure 8(a)). Consistent with the mRNA expressions, it revealed that after 7 days and 14 days of osteogenic induction, ALP and OCN were greatly downregulated in the *P. gingivalis*-LPS-stimulated group (*P* < 0.05) and significantly increased in a dose-dependent manner after incubation with IL-1ra (*P* < 0.05).

All the rGMSCs in different groups seemed to generate calcified nodules stained with ARS in a time-dependent manner (Figure 8(b)). However, rGMSC mineralization appeared to be downregulated in the treatment with *P. gingivalis*-LPS when compared to the control (*P* < 0.001). After 14, 21, and 28 days of cultivation, the formation of calcified nodules impaired by *P. gingivalis*-LPS was markedly enhanced by IL-1ra in a time- and dose-dependent manner (*P* < 0.01). All groups showed decreasing levels of calcified nodules compared to the control.

### 3.4. Activation of the NF-*κ*B Signaling Pathway in rGMSCs under Inflammatory Environment/Anti-Inflammatory Environment *In Vitro*

**Figure 9:**
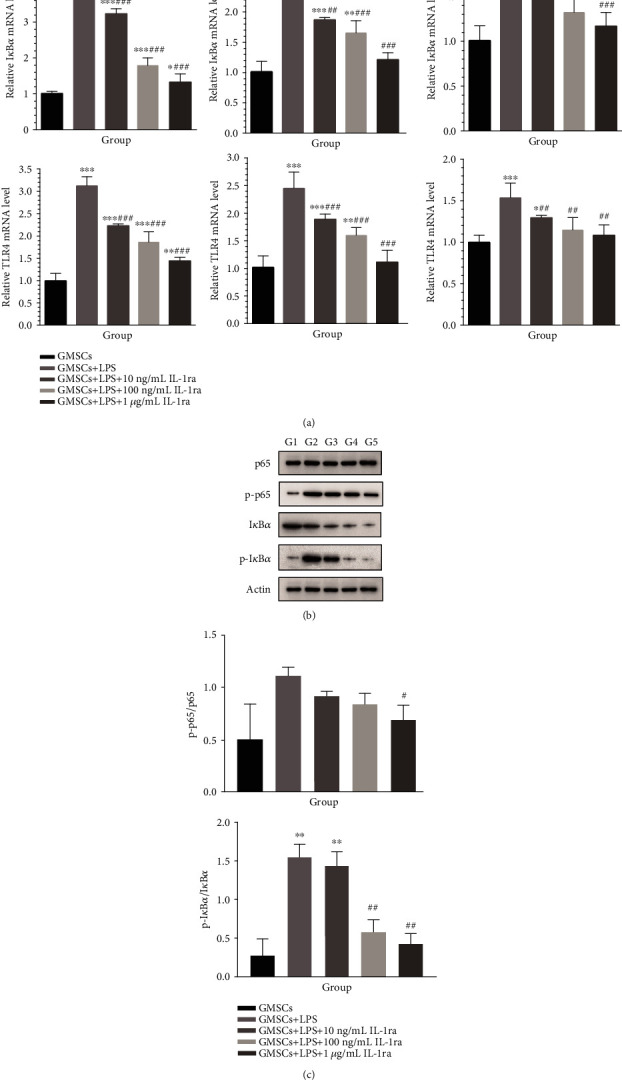
IL-1ra attenuates the *P. gingivalis*-LPS-activated TLR4-mediated NF-*κ*B pathway in rGMSCs. RT-PCR and western blot analysis were performed. (a) mRNA expressions of TLR4 and I*κ*B*α* were analyzed by RT-PCR at 1, 7, and 14 days. (b) Protein expressions of phosphorylated I*κ*B*α*, I*κ*B*α*, NF-*κ*B phosphorylated p65, and NF-*κ*B p65 after 1-hour induction in different groups (G1: GMSCs; G2: GMSCs+LPS; G3: GMSCs+LPS+10 ng/mL IL-1ra; G4: GMSCs+LPS+100 ng/mL IL-1ra; and G5: GMSCs+LPS+1 *μ*g/mL IL-1ra). (c) The ratio of p-I*κ*B*α*/I*κ*B*α* and NF-*κ*B p-p65/p65 in different groups. ^∗^*P* < 0.05, ^∗∗^*P* < 0.01, and ^∗∗∗^*P* < 0.001 vs. GMSCs; ^#^*P* < 0.05, ^##^*P* < 0.01, and ^###^*P* < 0.001 vs. GMSCs+LPS.

To investigate whether the NF-*κ*B pathway was activated in rGMSCs under *P. gingivalis*-LPS stimulation, we first examined mRNA expressions of the NF-*κ*B pathway-related gene I*κ*B*α* at 1, 7, and 14 days (Figure 9(a)). mRNA expression of TLR-4 which recognized LPS was also evaluated. Both I*κ*B*α* and TLR-4 significantly elevated when treated with *P. gingivalis*-LPS as compared to the control (*P* < 0.001) at all time intervals. On the contrary, rGMSCs incubated with IL-1ra obviously attenuated TLR4 and I*κ*B*α* expression in a dose-dependent manner.

To further confirm the activation and inhibition of the NF-*κ*B pathway, western blot was performed to evaluate the phosphorylation of I*κ*B*α*, I*κ*B*α*, phosphorylation of p65, and p65 (Figure 9(b)). Our results demonstrated that phosphorylation of I*κ*B*α* and p65 significantly increased in *P. gingivalis*-LPS-stimulated rGMSCs, while IL-1ra suppressed the phosphorylation of NF-*κ*B-65. As presented in Figure 9(c), ratios of p-I*κ*B*α*/I*κ*B*α* and p-p65/p65 clearly enhanced in the *P. gingivalis*-LPS-stimulated group, while they are decreased with increasing IL-1ra concentration. These findings indicated that IL-1ra demonstrated as an inhibitor for the *P. gingivalis*-LPS-induced TLR4/NF-*κ*B pathway.

### 3.5. Cytokine Release of rGMSCs under Inflammatory Environment/Anti-Inflammatory Environment *In Vitro*

**Figure 10:**
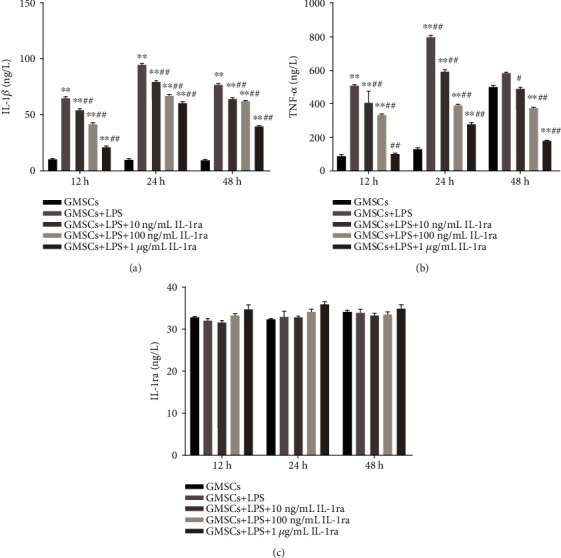
Effects of *P. gingivalis*-LPS and IL-1ra on cytokine release in rGMSCs after 12, 24, and 48 hours, as measured by using ELISA kits. (a) The production of IL-1*β*. (b) The production of TNF-*α*. (c) The production of IL-1ra. ^∗^*P* < 0.05, ^∗∗^*P* < 0.01 vs. GMSCs; ^#^*P* < 0.05, ^##^*P* < 0.01 vs. GMSCs+LPS.

As shown in Figure 10, the present study determined key cytokines released including IL-1*β*, TNF-*α*, and IL-1ra at 12, 24, and 48 hours. IL-1*β* and TNF-*α* significantly increased after *P. gingivalis*-LPS stimulation as compared to the control (*P* < 0.01), while pretreatment with the IL-1ra cytokine inhibited IL-1*β* and TNF-*α* release in a dose-dependent manner (Figures 10(a) and 10(b)). The most significant effects for *P. gingivalis*-LPS and IL-1ra on cytokine release were shown after 24 hours of cultivation. We did not observe any difference in IL-1ra levels between different groups (Figure 10(c)).

## 4. Discussion

Recently, protecting the osteogenic capacity of GMSCs in local inflammatory condition has aroused great interests. The results of the present study demonstrated that IL-1ra, as an anti-inflammatory cytokine to counteract the negative influence of *P. gingivalis-*LPS, protected GMSCs' cell viability and osteogenic ability and modulated inflammatory cytokine secretion. The underlying mechanism was detected as IL-1ra disrupting the TLR-4-mediated NF-*κ*B signaling pathway activated by *P. gingivalis*-LPS in GMSCs.

The microenvironment administrated the regeneration role of MSCs. Here, in our article, all the evidences combined suggested that *P. gingivalis*-LPS impaired colony formation, cell proliferation, migration, and osteogenesis abilities of rGMSCs, thus resulting in a deleterious effect on their tissue and bone regeneration properties, while IL-1ra was considered to play a protective role against *P. gingivalis*-LPS by reversing its negative effects. With the pretreatment with IL-1ra, GMSCs' original cell viability and osteogenic capability under inflammatory environment could be maintained in a time- and dose-dependent manner. In line with our results, present studies proved that LPS suppressed osteogenic differentiation and proliferation capacities in GMSCs [9]. It was also demonstrated that in conjunction with the IL-1ra-loaded HA hydrogel synthetic extracellular matrix (HA-sECM), the regenerative capacity of GMSCs could be enhanced in a typical periodontitis model in miniature pigs [13], which was consistent with our results in vitro.

Moreover, we underlined the inflammatory-resistant property of IL-1ra on *P. gingivalis*-LPS-stimulated rGMSC inflammation. Our findings showed that *P. gingivalis*-LPS could activate a positive feedback loop, the TLR4-mediated NF-*κ*B signaling, as demonstrated by the increase in TLR4 and I*κ*B*α* mRNA expressions and a surging of phosphorylation of the I*κ*B*α*/I*κ*B*α* ratio and phosphorylation of the P65/P65 ratio. The NF-*κ*B signaling pathway is noted as a strategic player in regulating inflammatory response. Zhou et al. [19] disclosed that via the NF-*κ*B signaling pathway, *P. gingivalis*-LPS upregulated GMSC proliferation potential, which is in line with our data.

We illustrated for the first time that IL-1ra rescued GMSCs from *P. gingivalis*-LPS-challenged environment by blocking the TLR4-mediated NF-*κ*B pathway. Inflammatory cytokines TNF-*α* and IL-1*β* induced by LPS are downstream of the TLR4-mediated NF-*κ*B pathway, which are both involved in the phosphorylation of NF-*κ*B [20]. In our present study, the upregulating synthesis of inflammatory factors TNF-*α* and IL-1*β* reached the peak after 24 hours of *P. gingivalis*-LPS stimulation, while pretreatment with IL-1ra partly eliminated their upregulation. The attenuating effect of IL-1ra presented in a dose-dependent manner, and the largest collapse of TNF-*α* and IL-1*β* occurred at 24 hours too. The attenuating effect on inflammatory cytokine production may make it possible for IL-1ra to protect rGMSCs' biological properties and osteogenesis capacity. Our results showed that *P. gingivalis*-LPS did not induce the secretion of IL-1ra. The observation is in contrast to the data published by Misawa et al. [21], in which *P. gingivalis* total protein extract- (*Pg*PE-) treated PDLSCs upregulated multiple inflammatory marker and chemokine secretion significantly, including monocyte chemoattractantprotein-1 (MCP)-1, IFN-*γ*, IL-6, IL-8, and IL-1ra. Various types of MSCs, stimulating factors, time, and load may be partly responsible for these controversial results.

However, our present study also has some limitations. For instance, the present study only used the selected pathogen *P. gingivalis*-LPS to mimic a periodontitis cell model, which is different from complex periodontal pathogens in a biofilm state in vivo. There are multiple ways to simulate periodontitis microenvironment in vitro. *P. gingivalis* [22, 23] and other periodontal pathogens including *Tannerella forsythia* (*T. forsythia*) [24], *Treponema denticola* (*T. denticola*) [24], and *Aggregatibacter actinomycetemcomitans* (*A. actinomycetemcomitans*) [22] interacted with host cells in some studies. Similar to our work, some other studies used LPS derived from *P. gingivalis* or other periodontopathogenic bacteria to incubate with host cells to characterize inflammatory condition [25–28]. Besides, some studies analyzed periodontal local tissues derived from patients diagnosed with periodontitis [29, 30]. Therefore, until now, the periodontitis in vitro model remains inconsistent. Our results from an inflammatory periodontal cell model in vitro should be taken cautiously when translating to an in vivo model. Due to the diversity of microenvironments in the context of periodontitis in vivo, further researches are clearly needed to determine whether IL-1ra could attenuate bone loss and contribute to tissue regeneration in human periodontitis lesions. Besides, GMSCs used in the present study were isolated from rats but not from humans. MSCs from different species may own intrinsic differences, resulting in different regeneration properties [31, 32]. Therefore, there remains a need to derive GMSCs from human to test our conclusion.

All in all, based on the achievements of the present researchers, our results further supported that rGMSC behaviors and osteogenesis differentiation potential impaired by *P. gingivalis*-LPS could be reversed under IL-1ra treatment. We revealed for the first time that the underlying mechanism may be IL-1ra downregulating the TLR4-mediated NF-*κ*B pathway activated by *P. gingivalis*-LPS. Thus, our work may provide some advantageous evidences for previous studies and future preclinical research practice.

## 5. Conclusion

Currently, this study highlights the protective effect of IL-1ra on rGMSCs in *P. gingivalis*-LPS-challenged inflammatory condition. IL-1ra promoted cell proliferation and migration ability and enhanced osteogenic differentiation of rGMSCs by inhibiting TLR4 overexpression and phosphorylation of NF-*κ*B under *P. gingivalis*-LPS stimulated condition. With increasing concentrations, IL-1ra rescues rGMSCs from impairment via downregulating inflammatory cytokine secretion. Altogether, our study points out a positive attitude for IL-1ra on attributing proliferation and osteogenesis in GMSCs affected by *P. gingivalis*-LPS. This study may provide a valuable theoretical basis for periodontal regeneration and novel therapeutic strategy in periodontitis.

## Figures and Tables

**Table 1:** Primer sequences for RT-PCR (supplied by Roche).

Gene	Forward primer (5′-3′)	Reverse primer (5′-3′)
ALP	GAGGAACGGATCTCGGGGTA	ATGAGTTGGTAAGGCAGGGTC
OPN	TGAGTCTGGAAATAACTAATGTGTTTGA	GAACATAGACATAACCCTGAAGCTTTT
OCN	TATGGCACCACCGTTTAGGG	CTGTGCCGTCCATACTTTCG
COL-I	ACGCTCAAGTCGCTGAACAA	TCAATCCAGTAGTCTCCGCTCT
Runx2	CAACCACAGAACCACAAGTGC	CACTGACTCGGTTGGTCTCG
TLR4	GAGGCAGCAGGTCGAATTGT	AGAAGATGTGCCTCCCCAGA
I*κ*B*α*	AACAGTCTGAACTCGCCACC	CACCAACCGCTCCTTCTTGA
*β*-Actin	AGTACAACCTTCTTGCAGCTCCTC	TGCCGGAGCCGTTGTCG

ALP: alkaline phosphatase; OPN: osteopontin; OCN: osteocalcin; COL-I: type I collagen; RNX2: runt-related transcription factor 2; TLR4: toll-like receptor 4; I*κ*B*α*: inhibitor of nuclear factor kappa-B alpha.

## Data Availability

The data used to support the findings of this study are included within the article.
